# Web AR Solution for UAV Pilot Training and Usability Testing

**DOI:** 10.3390/s21041456

**Published:** 2021-02-19

**Authors:** Roberto Ribeiro, João Ramos, David Safadinho, Arsénio Reis, Carlos Rabadão, João Barroso, António Pereira

**Affiliations:** 1 Computer Science and Communication Research Centre, School of Technology and Management, Polytechnic Institute of Leiria, Campus 2, Morro do Lena-Alto do Vieiro, Apartado 4163, 2411-901 Leiria, Portugal; eng.rob.ribeiro@gmail.com (R.R.); jr.joaoramos@outlook.com (J.R.); davidsafadinho.12@gmail.com (D.S.); carlos.rabadao@ipleiria.pt (C.R.); 2 INESC TEC, University of Trás-os-Montes e Alto Douro, Quinta de Prados, 5001-801 Vila Real, Portugal; ars@utad.pt (A.R.); jbarroso@utad.pt (J.B.); 3 INOV INESC Inovação, Institute of New Technologies, Leiria Office, Campus 2, Morro do Lena-Alto do Vieiro, Apartado 4163, 2411-901 Leiria, Portugal

**Keywords:** augmented reality, AR obstacle courses, human–drone interaction, marker-based AR, unmanned aerial vehicles, UAV control interfaces, UAV pilot training, Web AR

## Abstract

Data and services are available anywhere at any time thanks to the Internet and mobile devices. Nowadays, there are new ways of representing data through trendy technologies such as augmented reality (AR), which extends our perception of reality through the addition of a virtual layer on top of real-time images. The great potential of unmanned aerial vehicles (UAVs) for carrying out routine and professional tasks has encouraged their use in the creation of several services, such as package delivery or industrial maintenance. Unfortunately, drone piloting is difficult to learn and requires specific training. Since regular training is performed with virtual simulations, we decided to propose a multiplatform cloud-hosted solution based in Web AR for drone training and usability testing. This solution defines a configurable trajectory through virtual elements represented over barcode markers placed on a real environment. The main goal is to provide an inclusive and accessible training solution which could be used by anyone who wants to learn how to pilot or test research related to UAV control. For this paper, we reviewed drones, AR, and human–drone interaction (HDI) to propose an architecture and implement a prototype, which was built using a Raspberry Pi 3, a camera, and barcode markers. The validation was conducted using several test scenarios. The results show that a real-time AR experience for drone pilot training and usability testing is achievable through web technologies. Some of the advantages of this approach, compared to traditional methods, are its high availability by using the web and other ubiquitous devices; the minimization of technophobia related to crashes; and the development of cost-effective alternatives to train pilots and make the testing phase easier for drone researchers and developers through trendy technologies.

## 1. Introduction

**Figure 1:**
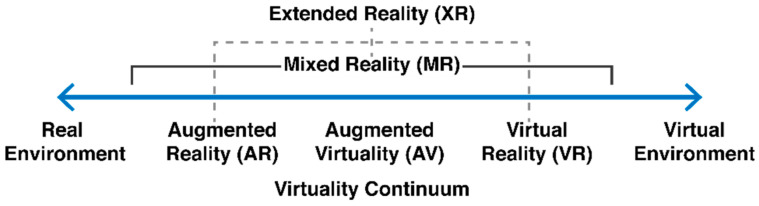
Adaptation of the Milgram and Kishino’s virtuality continuum [5].

The great advantage of unmanned aerial vehicle (UAV) usage includes the exploration of locations that are difficult to access and data gathering from an aerial perspective, which makes it useful in different areas and scenarios. The countless benefits and potential of drones are strongly related to their capacity to perform tasks that are difficult, dull, and dangerous [1], which is being intensively explored either for professional or routine activities [2]. Nonetheless, as stated in Cisco’s 2020 Global Networking Trends Report, immersive experiences powered with virtual reality (VR) and augmented reality (AR) are trending and emergent technologies [3]. Whilst VR allows the user to interact with an immersive and entirely virtual application, AR can present a layer of virtual content over a layer of real-world images captured in real time. The latter can help users improve their performance in many tasks by providing pertinent information about the pictured scenario. Mixed reality (MR) extends the concept of AR with features to detect changes in the real world, allowing the virtual elements to react and adapt, e.g., by simulating the collision of a virtual ball with a real wall [4]. VR, AR, and MR are represented by Milgram and Kishino through a virtuality continuum diagram [5], presented in Figure 1. Whilst VR, at the rightmost end, is represented by a fully virtual environment, AR remains closer to the experiences comprising a real environment. Additionally, MR brings the two ends of the continuum together, i.e., reality and virtuality. The three concepts are grouped in the general concept of extended reality (XR), which brings them all together [6].

This study is motivated by the need to simplify and improve drone pilot training and usability testing, thus decreasing technophobia and the exclusion that still exist regarding trendy technologies like UAVs and AR. The great majority of training alternatives for drone pilots are performed in entirely virtual simulators, which detaches people from the feeling of piloting a real drone with a real controller in a real environment. This motivates the combination of UAVs and AR to create training experiences that enhance the real world with virtual elements.

We identified and addressed the following issues:

- The control of UAVs and their flight dynamics tend to be difficult for new users to understand, especially for those who are not used to traditional remote controllers (RCs).
- Traditional AR experiences based on surface detection are limited to enthusiasts, since high-end hardware, such as HoloLens or Magic Leap 1, is required to run them. In the case of mobile devices, the oftware Development Kits (SDKs) for AR development are compatible with a specific list of devices which tend to be expensive, consequently reducing the number of users that can benefit from the technology in their daily lives.
- For UAV usability testing, developers usually set up simple scenarios and trajectories made of real obstacles. Every time a new trajectory is needed, the real obstacles (e.g., cones, poles, loops) must be moved. Though higher obstacles bring added value to tests of aerial maneuvers, it can be difficult and dangerous to change an obstacle every time a new trajectory is required, for instance, when it is placed on the top of a high wall.
- New users are intimidated and afraid to learn drone piloting in environments with real obstacles due to the fear of colliding and crashing [7], which reduces engagement in drone activities.

**Figure 2:**
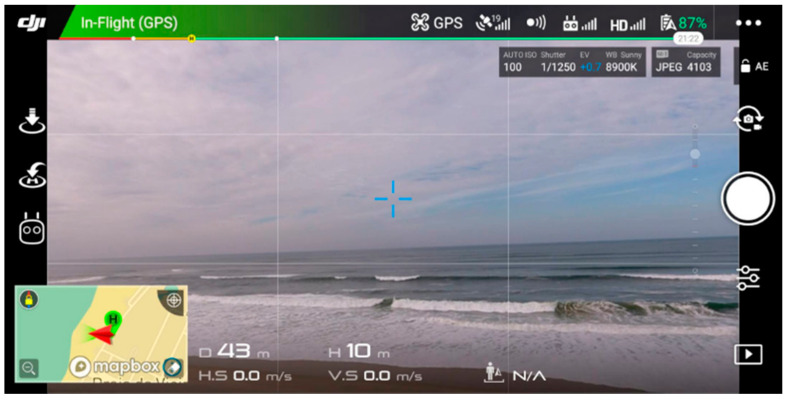
Screenshot of the DJI Go app that works as a mobile Ground Control Station during the flight of a Phantom 4.

These issues can be addressed through a dedicated solution that relies on web and AR technologies to create intuitive training experiences that can be represented in a browser, which are therefore available for a great number of users. The use of AR per se allows for the creation of different virtual training activities based on real environments and of digital obstacles which make it impossible to crash the UAVs. Because of these potential advantages, we propose a multiplatform cloud-hosted solution based on cost-effective technologies for UAV pilot training and usability testing. This solution includes the easy configuration of virtual sequences to build circuits with progressive levels of difficulty and introduces users to the control and behavior of drones. These circuits are intended to be used together with real-time control alternatives to create dynamic and interactive UAV piloting practice sessions. During the training session, the system uses AR to create a trajectory based on a reactive scenario, which is defined by a set of printed markers, placed in the real world. The drone’s camera is streamed in real time to a browser application that recognizes and interprets the markers that represent visual cues, made of 3D models or textual hints, creating a predefined virtual circuit. This means that the markers, which are physical objects, are placed in a specific position in the environment, either on the floor, walls, or ceiling, whereas their meaning is represented virtually and changes according to the configuration performed by the user. This way, through the placement of some markers around a training facility, the proposed system is able to generate several trajectories that require easier or harder tasks to be performed by the pilot, without the need for changing the obstacle in the real world. The resultant system is a data-driven controller that depends on a dynamic environment, which can change according to the placement of the markers around the training facility, and the digital system configuration, which can change the trajectory without moving the markers [8]. This way, this adaptive system is compatible with unknown environments and presents feedback according to the detected data and configuration [9]. Regarding the control, the use of technologies that are already understood by the common user, such as browsers, can facilitate the process. Web AR brings AR to a greater number of common users, considering that every device with a browser and a camera can theoretically run this kind of experience. It becomes possible to easily enjoy an AR experience through one of our personal devices, such as a computer or a smartphone, which means that this alternative does not demand that users buy additional ad hoc AR equipment or invest in an expensive device to use this kind of new and trendy technology. The pertinence of this technology is also related to the fact that mobile devices are already used as a ground control station (GCS) in commercial drones, for instance, in the case of company DJI (Figure 2) and Parrot. Besides showing the status of the aircrafts to the pilot, one of the functionalities of CGS applications is to display the drone’s camera stream. This way, web technologies can be combined with the GCS to represent AR experiences from the aerial perspective of a UAV and enhance the piloting experience, for instance, through virtual visual tips made with 3D (Three-dimensional) models and text.

Concerning technophobia, the placement of virtual obstacles around an open area can help decrease the fear of crashing the drone since it is impossible to collide with them. The training sessions become similar to the virtual UAV simulations, though in this case in a real environment, with a real RC and a real drone.

With this study, we intend to answer the following research question:

**Research Question:** “Can AR be used in a multiplatform solution, which is highly available and adapted for neophytes, to provide a way for anyone to learn and improve their UAV piloting skills, as well as to simplify the construction of scenarios to assess UAV research?”

To answer this question, we used the following methodology: First, we studied the basics of drone flight and piloting and the topic of AR, with examples related to drones, as well as Web AR. Then we conducted a review of studies related to applications combining UAVs with XR technologies and applications for UAV pilot training and assessment. Based on this knowledge, we designed an architecture to support an AR solution that can be used for UAV pilot training and testing UAV-related solutions. To validate it, we built a functional prototype that underwent different test scenarios, leading to improvements in the final system. The aim of this paper is to propose a solution that uses AR to potentiate UAV pilot training and the creation of scenarios for usability testing.

This study and the proposed solution present the following contributions:

- A reduction of the difficulty and intimidation involved in the process of learning how to pilot a UAV, using AR to provide context and feedback about the real environment;
- A reduction of the fear of crashing in a real environment through the use of AR to display virtual obstacles;
- A reduction of the effort, danger, and level of difficulty in the creation of flight circuits through the use of AR to display virtual obstacles;
- The application of Web AR technologies to create achievable multiplatform, highly available, cost-effective, and easy to configure AR experiences that work in real time;
- A combination of human–drone interaction (HDI) and AR to create an inclusive solution that potentiates pilot training and the execution of usability testing for anyone;
- The creation of real-world training courses with the real-time simulation of dynamic virtual obstacles through Web AR;
- Help for future research about UAV control in the assessment phase through the fast montage of aerial test scenarios consisting of AR markers.

This proposal considers two target user groups, pilots and researchers. With this proposal, neophyte pilots get a new tool to boost their learning process through a controlled experience with improved contexts and feedback provided by AR. At the same time, this proposal can reduce the level of complexity and effort for researchers who have to build personalized test scenarios in real environments. After placing the markers, the solution allows the user to create countless configurations and, therefore, different circuits.

The contributions of this proposal mitigate the factors that drastically reduce the engagement of common users in UAV pilot activities. About the technology itself, the use of Web AR reduces the limitations found in traditional AR solutions that usually require high-end devices and promotes the application of the proposed solution in several platforms. This also promotes user inclusion, since more personal devices can run the solution. This concept that considers new strategies for feedback and contexts using AR during the control of a UAV can potentiate the user experience and help pilots to read the real environment better with the addition of virtual elements.

If someone buys a drone and wants to train their skills with specific trajectories or environments cluttered with obstacles, they can print and place the markers and use this solution with their real drone. The cost of this approach is minimal compared to a reliable virtual simulation based on expensive VR systems like the HTC Vive or Valve Index (i.e., with prices that can go from USD 500 to more than USD 1000). Additionally, this solution can ease the acceptance of UAVs by technologically illiterate people or nonenthusiasts through their motivation to learn to pilot real drones without the fear of collisions, since obstacles are virtual in this solution. To the best of our knowledge, there are no proposals considering the development of a Web AR solution that takes advantage of visual markers to create virtual obstacle courses and integrate a UAV test framework or a dynamic hint-based circuit to train UAV piloting skills.

These contributions show the feasibility of combining HDI and AR to build a cost-effective training solution using visual markers, which opens many possibilities for future applications that can help boost multimedia experiences from an aerial perspective.

The proposed solution brings the following advantages besides its contributions:

- Improvement of training sessions in terms of practice and engagement;
- Use of trendy technologies to create interactive environments and flight trajectory feedback;
- Autonomous creation of configurable training scenarios for UAV pilots;
- Simulation of obstacles to train real scenarios without the possibility of crashing;
- Opportunity of using of low-range (i.e., cost-effective) personal devices to run the experience.

As for disadvantages, we find the following, which comply with the requirements for the proposed system to work:

- The piloted UAV must be equipped with a camera to stream its aerial perspective;
- The experience requires a device with a screen working as a GCS, besides the control interface.

Even though, in this study, we consider the solution to be used to train pilot skills for different UAVs, we focus the proposal and validation on drones.

The document’s structure is as follows: Section 2 presents an overview about drones and AR, as well as the related work about the relationship between UAVs and the emergent reality technologies, focusing on training and assessment research; Section 3 describes the solution and its architecture; Section 4 presents the development and evaluation of the functional prototype; and Section 5 presents the conclusions and prospects for future work.

## 2. Research Background

This research work explores ways to improve the way users interact with UAVs, whether they are neophyte pilots or researchers concerned about control improvements. Therefore, this section provides an overview of human–drone interaction (HDI) that summarizes the process of flight operation; an overview of augmented reality as a trendy technology that can enhance the feedback of the experience with virtual feedback; and a selection of studies that combine the previous concepts. Finally, we present the lessons learned during the research.

### 2.1. Human–Drone Interaction: Overview

UAVs can be useful in many fields and tasks. For instance, drones are used in surveillance to monitor road traffic in cities [10] or crowd supervision through facial recognition tools [11], and are currently used for beach monitoring in Portugal, allied to 5G mobile networks, to detect potential drowning situations. Autonomous deliveries are already being tested by retail companies [12]. There are applications for forest monitoring and fire prevention [13] and land pulverization for crop control in agriculture [14]. These aerial vehicles are also valuable tools for multimedia tasks like filming and photography, and there is a drone racing league that incorporates drones into the world of sports.

With regard to UAV flight operation, there are manual, semi-autonomous, and autonomous vehicles [15]. Only the latter are independent of a pilot, although this depends on several fail-safe systems to prevent accidents. In most cases, an in situ operator is required to control the UAV, which means that control must be learned and trained to get good results, accomplish hard goals, and prevent security issues [16]. The all-in-one solution to pilot the vehicles is the standard handheld RF remote controller, comprising a set of buttons, triggers, and joysticks.

UAVs can reach areas of difficult terrestrial access and sometimes there are cases where the pilot cannot monitor the position of the controlled aircraft, due to line-of-sight obstructions (e.g., buildings, rock formations). To solve this problem, UAVs stream their camera’s video to the control equipment for the pilots to safely fly them. The previous approach describes an exocentric perspective, which allows pilots to see what drones see through a video transmission displayed on the screen of the GCS (e.g., smartphone app, handheld remote controller, first-person view (FPV) headset), opening the possibility to create immersive piloting AR experiences. On the other hand, the traditional approach is based on an egocentric perspective, where the pilot monitors the drone flight by looking directly at it [17]. Comparing the two approaches, the benefits of the exocentric perspective are the monitoring of longer distances and the possibility of flying a drone in places of limited access.

### 2.2. Augmented Reality: Overview

AR is a trendy technology that can enhance the experience of controlling a UAV with new ways of providing a digital context and feedback over the representation of a real scenario. To understand the use of AR, this section summarizes its current state and provides examples of its application, approaches the way to create AR experiences and the technology required, and analyzes Web AR as an alternative compatible with several platforms.

#### 2.2.1. Context and Examples of Application

In July 2016, AR was introduced to the general public by the mobile video game Pokémon GO [18]. This type of application, where virtual graphics are superimposed over the real world is only possible thanks to improvements in mobile computing, built-in sensors, and cameras [19]. The advances of AR technology enabled the creation of large-scale experiences in indoor and outdoor environments, using different approaches [20]. For instance, Starbucks uses AR in advertisements with animations; the military uses AR to develop training experiences without using real ammo [21]; in industry, AR is used to present the repair instructions for the maintenance of machines; in the automotive sector, AR is used during the design phase to select car components from a specified virtual set [22]. Additionally, in gaming, in October 2020, Nintendo released Mario Kart Live: Home Circuit [23], a videogame for the Nintendo Switch console that allows the user to transform any living room into a kart track through AR and cardboard markers. During the game, the player can control a dedicated toy kart equipped with a camera through the console that displays not only what the camera streams, but also the virtual track elements. The technology employed in this commercial product is a remarkable example of what can be achieved with AR for remotely controlled vehicles.

#### 2.2.2. Achieving an Augmented Reality Experience

The groundbreaking part of an AR experience is not to place a virtual representation over the real world, but to identify a surface or a position to which the respective graphics can be anchored. This can be achieved through RGB-D (Red Green Blue-Depth) sensors, such as Microsoft’s Kinect or common RGB cameras, which are embedded in most mobile devices. However, the latter has limitations created by the environment, as Google lists in its AR Design Guidelines (e.g., reflective or single-color surfaces, dim lights, and extreme brightness) [24]. The use of a marker to define the position of a 3D model is one of the most common approaches, since it helps to overcome the problems related to single-color surfaces [25].

**Figure 3:**
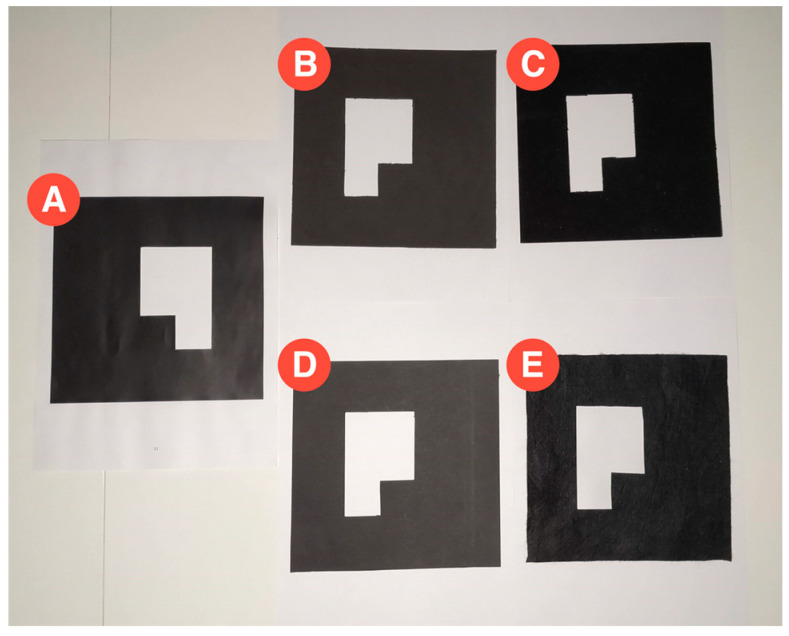
Comparison of five markers made of five different materials, under direct artificial white LED light. Whilst the marker (**A**) was printed, the others were cut and glued to standard printing paper. The other black materials are (**B**) EVA (ethylene-vinyl acetate) foam sheet, (**C**) velvet paper, (**D**) plain cardboard, and (**E**) felt sheet.

There are pattern-based markers, which depend on trained images, and barcodes that rely on a matrix that represents a number. To minimize the problems related to light conditions, the markers can be built with a more nonreflective material, instead of using a printed marker that easily creates light artifacts due to its reflectivity. After the comparison between five different black materials, which is represented in Figure 3, the printed marker is noticeably more prominent in creating light artifacts. The light source in this picture was an LED white lamp held over the markers and centered relative to them. Moreover, a visual analysis indicates velvet paper as the least reflective material of the five and, therefore, the darkest one.

Besides surface detection and marker identification, it is also possible to create AR experiences based on Global Positioning System (GPS), such as Yelp’s Monocle, which displays the location of restaurants near the user. Another example is Media Markt, a technology company that uses image recognition to display a virtual 3D model of the products over the store catalog [26].

#### 2.2.3. Equipment and Tools

**Figure 4:**
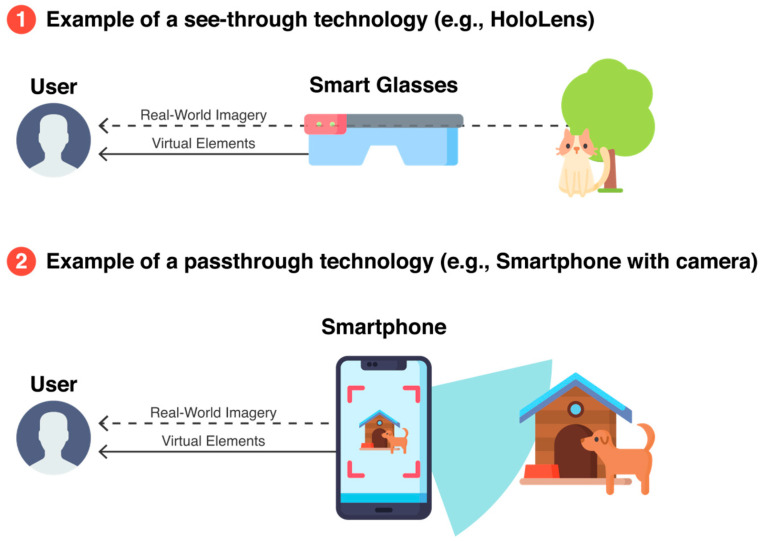
Representation of see-through and pass-through AR technologies.

To represent AR and MR experiences, there are see-through and pass-through technologies (Figure 4), with the latter also being known as video see-through [4]. Among see-through examples, we have the Magic Leap, HoloLens, and Moverio headsets, whilst pass-through live interactions with the surroundings can be experienced with smartphones, tablets, and VR hybrids. These hybrid headsets correspond to VR head-mounted displays (HMDs) with a camera, such as the HTC Vive Pro [27]. The main difference between the two is that pass-through relies on a camera and a conventional screen to display the combination of the captured imagery with computer graphics, whilst in see-through, the users can see the real world directly. This example occurs with HoloLens, which is a pair of smart glasses that includes holographic projectors to represent the virtual visuals over what we already see.

#### 2.2.4. Web Augmented Reality (Web AR)

Besides game development for standard VR equipment (e.g., Sony’s PlayStation VR, Oculus Facebook’s Oculus Rift) it is also possible to create both VR and AR experiences through the frameworks of game engines like Unity and Unreal Engine with specialized SDKs (e.g., ARKit, ARCore, Vuforia, EasyAR) [28]. However, to run the built applications, these SDKs require compatible devices with specific hardware, which makes the expansion to different platforms more difficult. Web AR can solve this problem [29].

The Web AR concept relies on web technologies to run an AR experience in a browser application, supposedly making it compatible with every device with enough computing capabilities [30]. There are different classifications regarding the browser’s role in the computational tasks [31], each one with their limitations, as presented in Table 1. The pure front-end approach includes a JavaScript engine at the users’ side that performs all the algorithms and calculations. Due to JavaScript’s performance limitations, there are browser kernel-based extension solutions that consider a deeper browser integration to get more powerful resources. However, this reduces the cross-platform capacities of these solutions since different browsers may have specific SDKs and different functionalities. To reduce the dependency on the users’ devices regarding complex calculations for the AR algorithms, the final approach combines the browser with cloud services. This synergy delegates all the processing to the cloud. The downside of this method is the latency of the network communications, a problem that should be minimized in the near future through the introduction of 5G wireless networks that are expected to bring lower latencies (of up to 1 ms).

This technology is the subject of research in different areas. In [32], the authors developed a study to help Chinese traditional handicraft students to improve their skills in painting Cantonese porcelain. The handheld device acts as a virtual brush, while its camera points to a marker displayed on the porcelain itself. In [33], the authors use the JavaScript 3D library three.js to create a web application that performs the reconstruction of a 3D scene, through the placement of virtual models, based on the imagery captured by the camera of the device. Web AR is an easy alternative to extend existing applications with AR functionalities. For instance, the WeChat messaging application embeds this technology to enhance the users’ experience through immersive involvement [34].

### 2.3. Related Work

The documents reviewed in this section were selected according to the results delivered by Elsevier’s Scopus citation database. The search query submitted to the database form was “TITLE ((uav OR drone OR quadcopter) AND (((control* OR pilot* OR simulat*) AND (train* OR improve* OR education OR test* OR assess* OR evaluat*)) OR (“augmented reality” OR ar))) AND PUBYEAR > 2015”, which returned 246 articles. These results were analyzed and, from the total, we selected 16 papers that were relevant for the scope of this research work. The following sections present and discuss these papers.

#### 2.3.1. Applications using Unmanned Aerial Vehicles and Extended Reality

Beginning with VR, Mangina et al. [35] proposed the integration of a VR system with a low-cost semi-autonomous UAV, with the objective of immersing the user in the environment of the aircraft, providing visual content to people with limited mobility. This study combines the robot operating system (ROS) and the AR drone to provide the required features, such as the transmission of control data and video feed. In addition, the motion sensor of the Oculus Rift HMD generates data that make it possible to pilot the drone with six degrees of freedom (DoF), using head movements. The speed and altitude of the drone are controlled with a Wii Nunchuck controller connected to an Arduino Uno. The connection between the laptop running the system and the UAV is established via Wi-Fi to send the control data to the drone and receive the video feed to display on the HMD. This work was tested by 10 participants that described the interaction with the VR system as intuitive, but harder to use in tight spaces. In their work entitled DroneVR [36], Nguyen et al. used web technologies to create a VR environment reconstructed with real-world fly data and simulate the control of a UAV to present a higher perspective of 3D path planning to pilots. Besides the manual navigation of a UAV, the simulator includes functions such as automated routing to find the optimal path among the mission’s locations, a safe return to the home point, and object detection and tracking. This experience was implemented with JavaScript libraries, e.g., the three.js 3D library, which enables the creation of a browser-based VR application, not limited to a single device such as the applications developed for traditional VR kits like the HTC Vive. The real-time geographical data to build the virtual scenery were obtained from OpenStreetMap.

Regarding AR, in [37], Manzoli et al. proposed an interesting approach to aid boat navigation during foggy weather or at nighttime, relying on AR to render the coastline through data collected by a 360° camera attached to a drone. As with what Google did to create Street View navigation, the objective of this team was to record rivers and coastlines for water navigation. The process of imagery collection is challenging. One of the challenges is the natural instability of the water, whilst the other is the necessary flight time to record the scenery with a UAV. This last problem was addressed using a tether-powered UAV, connected to the boat by a 45 m power cable. After the collection, the panoramic images were treated by stitching software to join the pieces of the scenery together. Although the solution was not fully implemented, this research proposes the AR rendering, not only of the coastline, but from the nearby boats as well, through the connection to an automatic identification system (AIS). Unal et al. [38] modeled an estimation of the Roman Bath of Ankara, an important archeological site in Turkey, which is almost in ruins, to virtually preserve the cultural heritage of the place. The experience consists in tracking a UAV through GPS and orientation sensors to simulate the flight around the real place. According to the UAV position and rotation, the virtual model is represented over the drone’s camera. In a later paper, Unal et al. [39] coined the concept of distant AR with a work that intends to improve the presentation of cultural heritage through AR using drones. In the previously presented geolocation system, the authors included the imagery of a monocular camera to perform vision-based scene tracking, using a DJI Phantom 3 Professional drone. In this work, the GPS and gyroscope modules integrated in the drone were used for the geolocation tracking, based on several recordings of predefined flight trajectories. The geolocation data were filtered and converted to 3D cartesian coordinates, in order to present the AR experience correctly. To calibrate the drone’s camera for the vision-based tracking, the authors used the Matlab Camera Calibration Toolbox and a rectangular grid. The Emgu CV.NET wrapper enabled the use of the OpenCV framework with the Unity game engine for purposes of object tracking, and the Perspective-n-Points technique was employed to estimate the position of the camera. The 3D model of the archeological reconstruction was built with 3D Studio Max, which was improved in terms of efficiency using the MultiRes feature to delete unnecessary vertices. Chen et al. [40] used AR to create an interface to generate drone trajectories through a mobile application and the camera of the device running it, that captures the active drone through an egocentric perspective. With this interface, users can touch the screen of the device to point at the location they want the drone to reach or draw a pattern to follow as a trajectory. To calculate the position of the drone in the scene, the team used the OpenCV Acuco algorithm, and the AR elements were implemented through the ARkit AR development platform for iOS devices.

Liu et al. [41] proposed a solution to integrate the flight of a UAV and AR in building information modeling (BIM) projects to synchronize the inspection of a building of interest with the presentation of the virtual model. The authors used a fast coordinate transformation algorithm to convert the coordinates of the real UAV to the equivalent position in the BIM project, allowing them to watch the real inspection video at the same time as the virtual building model. The prototyped system behaved with submeter precision, leading to easier retrieval of information, increasing the efficiency of the process. Dam et al. [42] explored the topic of civil infrastructure inspection using semi-autonomous drones and AR, through a VR simulation of a bridge inspection. In the proposed platform, AR is used to present visual cues that can improve the detection rates by the operators. Four types of visual cues (i.e., none, bounding box, corner-bound box, and outline) were tested by 28 participants with the VR simulation running on an HTC Vive Pro HMD. The results showed that the corner-bound box is useful to attract the attention of the user in a cluttered visual scene. Pavlik [43] presents a paper that studies the great potential of drones in the creation of immersive news content. The four factors that support this potential are FPV perspective, geo-tagged audio and video, volumetric 360-degree video and 3D audio, and the generation of novel content types, like photogrammetry. The combination of UAVs with VR and AR plays a significant role in the creation of novel content types. For instance, spatial journalism, where the viewer experiences an immersive video report (e.g., warzone, on-stage music festival), or immersive storytelling experiences based on several drone-captured streams that enable multiple viewpoints of the same video report.

As for MR, Llasag et al. [44] studied how this technology and UAVs could be combined to create a human detection system for search and rescue operations. The team proposed and implemented an architecture composed of a mobile app and an MR app, connected by a central communication server that saves the system’s configurations. The mobile app is responsible for running the deep learning human detection algorithms over the video stream captured by the drone. Then, the results of this processing are transmitted through Transmission Control Protocol/Internet Protocol (TCP/IP) to a HoloLens headset, running the MR app. The holographic projection rendered by the headset corresponds to the combination of a virtual map with the FPV of the pilot and the aerial view of the drone. If a human is found, their detection is represented in the holographic rendering of the drone’s camera. This implementation used a DJI Phantom 4 drone, which transmits its video and telemetry to its RC. Furthermore, this data can be accessed by the device running the mobile app through a Universal Serial Bus (USB) connection to the RC.

#### 2.3.2. UAV Pilot Training and Assessment

Bruzzone et al. [45] presented a study where the objective is to simulate a disaster in a chemical plant to train UAV pilots for emergency situations. The authors present the drone as a tool to verify which chemical substances were released, the inherent risks and propagation state, and the health condition of the people around the scene, if any. In the simulation, the operator can configure the aircraft with different sensors and autonomy, according to the disaster scenario. After the training session, the system provides feedback concerning the operator’s performance, considering metrics such as the mission duration, the battery level, the coverage area, and the triage efficiency, among others. The participants that tested this training solution showed certain improvements, such as the reduction of the number of mistakes and collisions and, consequently, a reduction in the duration of the mission. Keeffe et al. [46] used the ROS as the basis for an application to train drone pilots. In this work, the ROS is combined with the SDK of the Parrot AR drone to manage the different modules with respect to the control of the respective UAV (e.g., navigation, video stream). The altitude control is performed with a Wii Nunchuck and the camera view of the drone is displayed in an Oculus Rift headset that also controls the rotation of the drone. The 2D video captured by the UAV was transformed in a stereoscopic 3D representation with the Oculus Rift SDK and OpenCV being used to display telemetry information over the real recording. The 20 participants that evaluated the system were divided into three different skill groups. The group with no experience could fly the drone with minimal help. This study is a follow-up to a previous one [35]. Zheng et al. [47] designed a semi-physical simulation for a UAV training system, with the goal of simulating the ground control and the airborne activity, to improve emergency response capacity. To do so, the team developed a mathematical model for the UAV based on aerodynamics and airfoil parameters, as well as the mathematical simulation for components, such as the navigation and the flight controller. The environment implemented with VC++ and OpenGL displays the scene according to the data coming from the simulated systems. To train the operator, this simulator also includes modules to build and configure training courses with different conditions, such as air route, and to evaluate the flight performance based on specific rules. Another UAV flight training simulation was created by Liu et al. [48] to better educate trainees through a VR environment. The VR application was developed with Unreal Engine and the Visual Studio IDE. It runs on a computer, uses a VR HMD for the display and a pair of handles for the control. Three modes were developed: the training mode, which is audio guided so that trainees get familiarized with flying maneuvers; the competition mode, which consists of flying over the obstacles within a venue to obtain the best score; and the entertainment mode, which offers a 360° view to participate in the assembly of different drone models. An interesting perspective of UAV flight tests is given by Zheng et al. in [49], which proposes a design for a simulation test platform for a UAV control system. In this design proposal, the authors consider a dynamic simulation of a normal scenario and another with fault injection to replicate the random faults that can happen in a real-world situation. The suggested guidelines are defined according to the subsystems that constitute the UAV (e.g., flight control, sensorial modules) and are aware of the environment through the simulation of different wind conditions and of the aircraft’s characteristics by simulating its characteristics. Go et al. [50] created a system that enables the interactive training of FPV drone piloting, using an MR approach consisting in a virtual environment and a real drone. The system provides an interface for the trainer to design the virtual scenario and manage the training session using an HMD. On the other hand, the pilot role runs the experience remotely in an empty area to avoid colliding the real drone during the control through the personalized virtual environment. Regarding the used technology, for the training design module, the authors used the Unity game engine and the Photon Unity Networking framework and Photon Voice service to enable the interaction and communication between the users with different roles. The drone used by the pilot was a DJI F550 hexacopter equipped with an Nvidia Jetson TX2 AI computing device and a Stereolabs ZED Mini stereoscopic camera. As for the experience itself, whilst the trainer designs the circuit with a desktop computer, wearing an HTC Vive Pro HMD [51], the pilot carries an HP Omen VR Backpack computer and wears an Oculus Quest HMD.

### 2.4. Lessons Learned

The related work shows that researchers are interested in obtaining the best from immersive reality to apply in different UAV case scenarios. One of the main goals is to combine a real UAV with a virtual replica of a specific scenario or activity in an application that enhances the perspective of the pilot over the task in hand in real time, improving the pilot’s awareness of the location of the drone and, therefore, the precision of the control. AR and MR are used to add a layer of graphical information over the real world that can be about the UAV or the environment captured by its camera. The review of the literature provides relevant insights for our current work, regarding how UAVs and the reality spectrum are combined, as well as the training methodologies and the approaches used to evaluate them. This is an interesting combination due to the aerial view that is now easily achievable and used to obtain new perspectives, either for cultural (e.g., simulated representation of an ancient location) or professional purposes (e.g., building inspection). Another relevant observation is found in [42], a paper where the authors concluded that visual cues help in attracting the attention of users. For UAV pilot training, the most popular alternatives are flight simulators, which try to virtualize the control as realistically as possible, so that pilots are trained and get used to the real flight mechanics and can be able to deal with malfunctions or hazardous encounters. There are other approaches not entirely virtual, like the one presented in [50], where the pilot controls a real UAV while seeing a virtual circuit through an HMD. However, the equipment required to run the experience is very costly, which is a problem when the motivation and goal of this paper is to bring UAVs to as many users as possible using their personal devices.

As part of this study, we intend to propose a solution that implements a new concept that relies on the use of AR to bring new contexts and feedback to pilots and UAV researchers. The goal is to improve pilot training and decrease the effort needed to prepare a test scenario in real environments, with real UAVs, but with virtual obstacles and goals instead. There are few papers in the literature that combine the real and the virtual worlds through AR, or MR, and web technologies, and to the best of our knowledge, there is no AR application for UAVs with the objective of creating virtual courses over the real world to train UAV pilots or help researchers or developers assess their prototypes. These arguments present a great opportunity and support for the development of a solution that exploits AR and web technologies to improve the perspective of the pilot over the control of a UAV.

## 3. Proposal of the Solution

There are problems in the control of UAVs that affect both neophyte pilots and researchers. Some of them relate to the difficult learning process and others to the preparation of a scenario to practice or assess research work related to HDI. Considering these problems, the current paper proposes a solution that intends to improve the engagement of anyone in UAV piloting and decrease the complexity and effort found in the creation of a personalized flight scenario. This section presents the description of the proposal, the architecture to support it, and the implementation of the solution.

### 3.1. Description

This proposal is the first step of a complete solution to help both piloting novices and developers or researchers working with UAVs. This work is not about the development of a control solution. Instead, it proposes a solution to complement a control session in real time, where the environment is real, but the obstacles or trajectory indicators are virtual.

**Figure 5:**
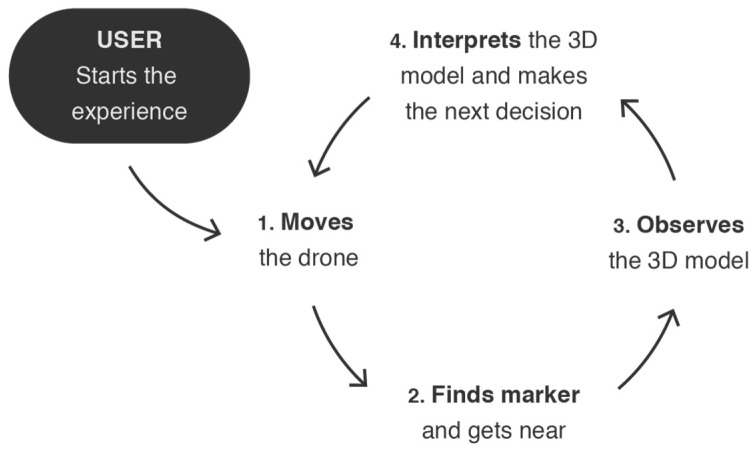
Feedback loop of the application representing the actions of the user.

Therefore, this solution focuses on two topics. One of them is to facilitate the process of learning how to fly manual and semi-autonomous drones or improving piloting skills. The other is to create an easy-to-use obstacle tool to test the research and production of UAV solutions, including new controllers or intelligent systems for autonomous flight tasks, such as obstacle avoidance or flight path calculation. We propose a system to generate virtual courses, made of virtual points of interest (POIs), which can be adapted to constitute activities resembling a common UAV obstacle course, a guided tour, or a treasure hunt activity. This system can act as a step-by-step method for introducing new commands and maneuvers to the users, or simply to produce hybrid courses (i.e., made of real environments with virtual elements) for researchers and developers to perform evaluation metrics. The POIs of the courses are identified through AR markers, which are printed and placed over a predesigned scenario. The user is guided through a sequence of markers ensured by dynamic representations that are virtually placed over each marker, according to the specific circuit. The use of markers and their respective detection allow for the system to keep track of the progress of the pilot. For instance, the pattern of each marker represents an integer value that is used as an identification number, which estimates the location of the aircraft, according to the configuration of the sequence and the detection event. This detection event can also be used to count flight times, such as how long a pilot takes to arrive at a specific marker or to finish the circuit. This approach facilitates the time-counting process and makes it unnecessary to integrate location or detection technologies, such as GPS or proximity sensors, both in the drone and in the real POIs or obstacles, if relevant. With the same scenario and markers, it becomes possible to create several different trajectories. The solution can configure dynamic sequences based on each marker’s identification number to define levels of progressive difficulty. The proposed approach can digitally change the circuit (not only the order of the sequence but also the meaning of each POI), without changing the placement of the markers. The same would not happen with real objects. Moreover, the system can change the interpretation of each marker to comply with different sequential challenges. For instance, in a training activity inspired by a treasure hunt, a marker that in a certain circuit was the last POI, in another one can act as an intermediate step of the trajectory and suggest that the pilot move right to find the next POI. Briefly, the virtual course application is represented by the feedback loop in Figure 5. The user starts the experience and moves the drone to find a marker. When a marker is found, the users get close, observe the 3D model represented on the marker, and interpret its meaning, so that they can move the drone in the right direction. The loop continues until the end of the experience.

Although the two modes converge as training activities, they were developed considering different needs. Whilst the mode inspired by a treasure hunt was considered for users to learn different sequences of piloting tasks through hint-based trajectories, the simple obstacle course was thought of as an improvement for developers that want to test the usability of their UAV solutions with the same conditions and define a low-cost scenario with printed markers. With these approaches, we aim for a solution that can help the participants improve their control skills, in terms of dexterity and keeping track of the direction the drone is facing. In a general way, as Table 2 shows, the two modes are constituted by different markers, are intended to perform different tasks, and are built considering different goals.

Whilst the treasure hunt presents arrows pointing the way, so that the users can follow a trajectory, the obstacle course presents one or more types of obstacles (e.g., cones or hoops), so that the users must dodge the hazards to avoid a virtual crash. In the case of the treasure hunt activity, the goals are to learn the basic flight mechanics and improve maneuverability and the capacity to follow well-defined paths as fast as possible, whilst the goals of obstacle courses are to improve reaction time and the decision capacities of the user to plan a new trajectory.

### 3.2. Architecture

**Figure 6:**
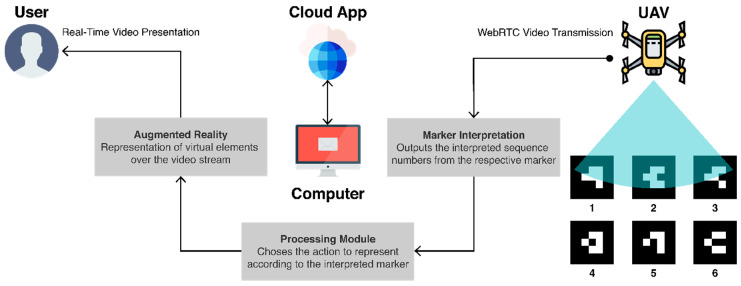
Architecture of the solution, which depends on three separate modules responsible for interpreting the visual markers, processing their meaning, and representing the AR experience.

The architecture of this solution is composed of the user, a drone with a camera, and a computer connected through the Internet to a cloud platform, as presented in Figure 6. It represents an activity where a user pilots a drone while looking at a computer running an AR browser application based on the drone’s video stream and graphic elements that create a virtual obstacle course. This application is loaded from a cloud platform where it is hosted. The architecture assumes that the UAV can establish a connection to a network, either local or via the Internet, which is necessary to run the service responsible for broadcasting the camera of the UAV in real time and wirelessly. To avoid jeopardizing the performance of the drone, the AR experience’s processing module was created in the web app that runs on the client’s end, in the device working as a GCS (e.g., computer, smartphone), which is responsible for obtaining the video stream and showing it to the user. Before reaching the user, the video stream passes by a marker interpretation module that periodically checks for AR markers. If it detects a marker, a message is sent to the processing module, which is responsible for interpreting it and deciding on the correct action, according to the current meaning of that marker, which can depend on the sequence and its status. Finally, the action is specified by the AR module through the representation of a virtual visual cue, made of a 3D model or a textual hint, over the video stream placed on top of the detected marker.

**Figure 7:**
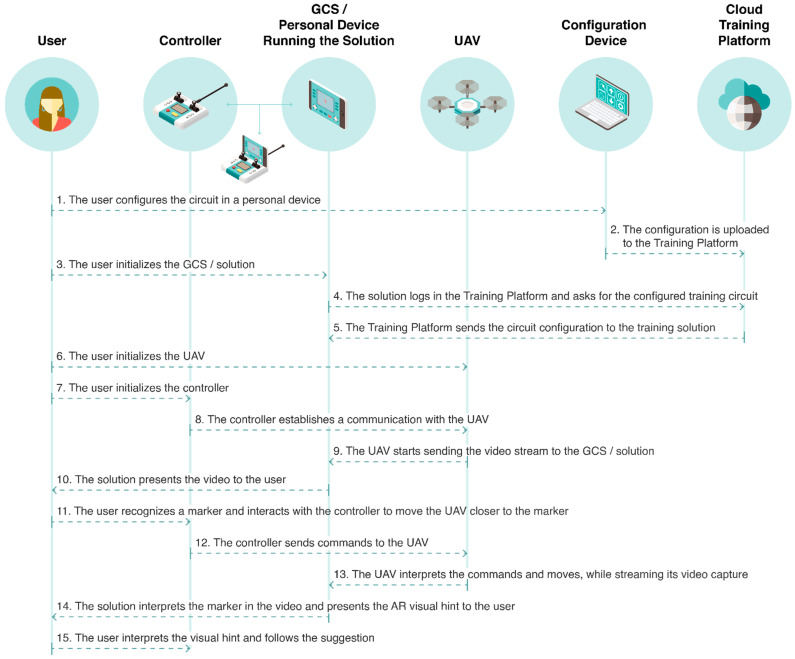
Architecture for the full training system for UAVs (Unmanned Aerial Vehicles), representing the integration of the solution with the control layer.

This architecture represents a solution to be used as a new data-driven feedback layer over the control layer. The integration of the solution in the control layer and its parameters is not described in detail, as the focus of this work is the validation of an AR solution to potentiate the control, and therefore training activities. A proposal for the architecture that describes the integration between the controller and the proposed solution is illustrated in Figure 7. This architecture presents a vision for the solution to work as fully integrated in the control and considers that both the personal devices (i.e., GCS and configuration device) have an Internet connection. This integration facilitates the transmission of data between the entities that participate in the control system, i.e., the controller, the personal device running the solution and working as a GCS, and the UAV itself. Therefore, the UAV transmits its video and parameters (i.e., flight stats and control) to the controller. The data, required by the GCS proposed solution, to monitor the flight and execute the training tasks, are then obtained directly through the connection that merges the controller and the personal device as a single entity. This approach is similar to the one adopted in current commercial drones, like the DJI Phantom 4.

### 3.3. Implementation

**Figure 8:**
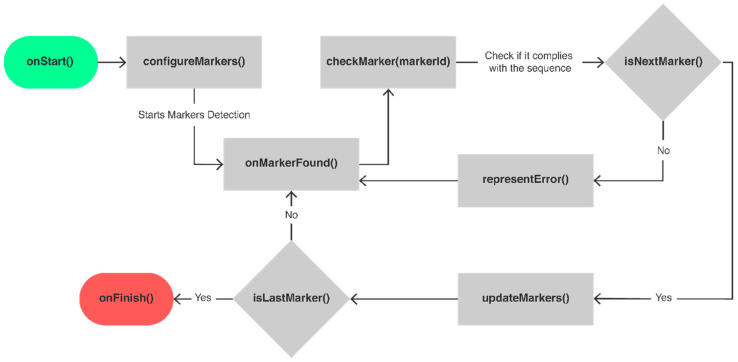
Simplified logic cycle of the implementation, which describes how the experience works from start to finish. The round shapes represent lifecycle events, the rectangles the methods and functionalities, and the diamonds a Boolean conditional statement check.

There are implementation differences depending on the system modes. Whilst in an obstacle course every marker is configured as an obstacle, the treasure hunt mode follows a sequence that is updated in real time as the UAV approaches a marker that corresponds to the correct next index of the current sequence. The logic for the implementation of the treasure hunt mode is presented in Figure 8, which shows the dynamic representation of each marker throughout the progress in the level. The logic begins with the configuration of the markers that constitute the trajectory of the level. Considering that each marker represents an identification number interpreted as an index of the POIs that define the trajectory, this configuration corresponds to the creation of a sequence and attribution of meaning for each of the markers (e.g., go left, increase altitude). With the end of this task, the detection of the markers starts. When a marker is found, the system uses its identification number to check if it corresponds to the next element in the defined sequence. If so, the system updates the meaning of the markers to correctly represent the sequence. Otherwise, it shows a symbolic error notification to the user, like a red cross 3D model. After the update, the system verifies if the marker is the last waypoint of the trajectory. If not, it continues the detection loop. If it is the last one, the experience ends.

There are four states for the markers: Invalid, Validated, Current and Next. Each time a marker in the Next state is detected, the previous marker changes its state to Validated. This marker is defined as Current and the next one is defined as Next. This way, each marker must have references to the previous and the next ones. The remaining markers are identified as Invalid. The meaning of each state is summarized in Table 3.

Besides, a marker must know the direction of the next one, to display an arrow for the user to follow correctly. The direction is represented with an integer value, from 0 to 7, which corresponds to the cardinal directions: north, northeast, east, southeast, south, southwest, west, and northwest. The negative values indicate that there are no previous/next markers. Each marker is also classified as first, default, or last to describe their role in the level. This represents different graphics on the start and finish markers to determine if the level is about to start or end, based on their representation.

## 4. Development of the Prototype

The prototype built in this study follows the proposal and its implementation to validate the concerns of this paper, which are to provide a way to improve piloting skills and to assess UAV prototypes created by investigators or developers. Therefore, this section includes the description of the prototype and its evaluation.

The control of a real UAV is not included in the developed prototype, since the focus of this work is the development of a solution complementary to the control, based on the enhancement of real-world images captured in real time with virtual elements.

### 4.1. Description

The solution developed in this study uses web-based technologies, so it can be used in the largest number of compatible platforms possible, with the unique implementation of a cloud-hosted web system. Every device with a web browser and a camera should be capable of running this prototype, unlike AR development platforms, such as ARCore or Vuforia, which require devices with special support. To simulate the operation of a modularly built UAV with an open flight controller, we prototyped a portable system made of a Raspberry Pi 3 model B small computer, powered by a power bank, which streams the video feed of its Raspberry Pi camera through Wi-Fi to the real-time browser application, in order to create a pass-through AR experience. A computer is connected to the same local Wi-Fi network as the Raspberry Pi and the video stream is transmitted through WebRTC, which enables real-time communication with the camera of the drone through a web browser. To build the browser application, we used HTML5 to create the GUI, and JavaScript to implement the functionalities. The libraries AR.js [52] and A-Frame [53] enabled the development of the required AR features, using barcode markers, which were the detection of a surface and the representation of virtual 3D models over the real-world layer, relative to that surface. AR.js is supported by the ARToolkit SDK, which uses a segmentation algorithm based on a grayscale threshold that is used to generate straight contour lines that are used for marker detection [54]. In this case, the real-world images are captured by the Raspberry Pi’s camera. Six barcode markers were considered and previously built according to a 3 by 3 matrix pattern. These markers work as placeholders for the virtual 3D models that represent the phases of the training trajectory, according to the predefined sequence. This prototype works with the two proposed modes, which are the treasure hunt and the obstacle course.

**Figure 9:**
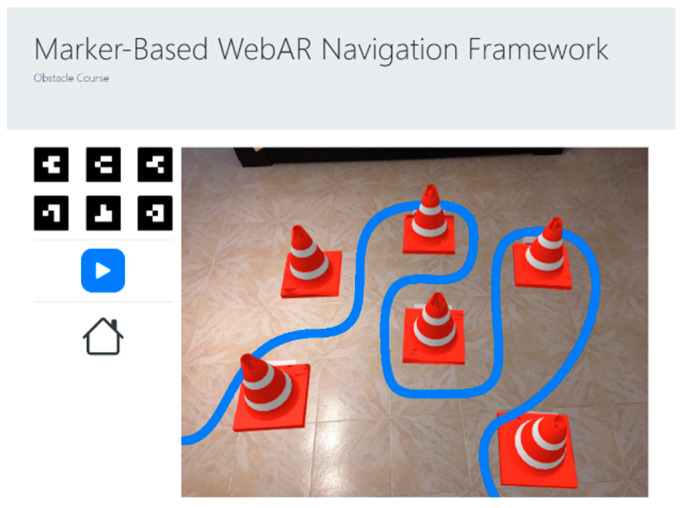
Simple obstacle course, where the objective is to contour the cones (the blue line was added later as an example of a trajectory).

The treasure hunt circuit is made of four models that describe the status of each POI. A green check model shows that the POIs were already visited in the correct order, whilst a blue arrow is shown in the current marker to point in the direction of the next one. When the player visits a new marker in the wrong order, a red cross is presented. Finally, the last marker presents a checkered flag to show the user that it is the end of the trajectory. The obstacle course was implemented considering a sequence of markers distributed parallel to the ground to create an obstacle course made of street cones. The goal is to define a flight path for the pilot to accomplish, as in Figure 9.

### 4.2. Assessment and Improvements

The development of the prototype was divided into iterations that helped improve the result. The assessment was separated into two phases. In the first phase, the solution was tested to validate the implementation logic and the success of the intended functionalities. Then, the performance of the prototype itself was evaluated to measure the quality of the experience built with Web AR. This section presents the two assessment phases and their results.

#### 4.2.1. Validation of the Solution

**Figure 10:**
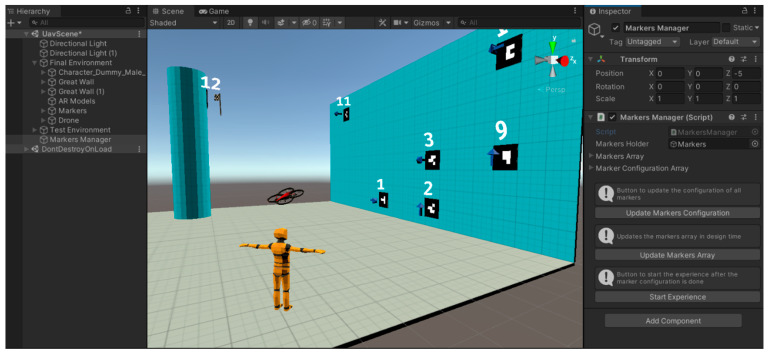
Screenshot of the early prototype developed with Unity.

The validation of this solution began with the development of a virtual simulation of the proposed solution using the Unity game engine (Figure 10). The objective of this simulation was to represent the full concept of the solution and implement and test the logic required to generate an activity inspired by the treasure hunt and obstacle course modes. In this virtual experience, we simulated the flight of a drone, as well as the control, the placement and configuration of the available barcode markers as a sequence, and their detection using a camera integrated in the virtual drone.

**Figure 11:**
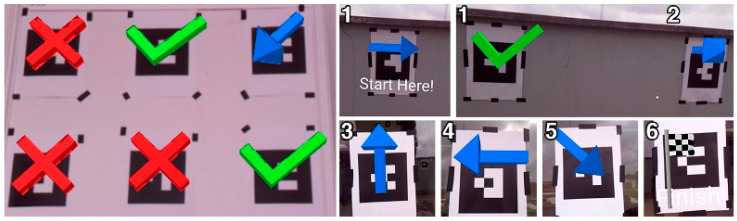
Markers on a 2 by 3 grid (left) and an example of a sequence performed in the correct order in a real scenario (right).

To evaluate the operation of the proposed solution, we created several distinct scenarios with 6 barcode markers, in which the first step was to build the sequence and define the level with the following order: 2, 6, 3, 5, 1, 4. The first scenario corresponds to a 2 by 3 grid, displayed on a single wall, as shown in Figure 11, which shows the unfinished level, where only three markers were visited. The user should move to the 4th marker, identified by the barcode value 5, according to the sequence. As the arrow over the 3rd marker shows, the next point is located southwest. The first scenario led to improvement related to the better placement of the virtual models, detection angles, and distances. After validating the functionalities, the second and more complex scenario was built using the same markers placed along the interior side of two parallel surfaces, creating an experience dispersed throughout a 3D space. During this assessment, the prototyped system, replicating a UAV, was moved along the sequence represented in Figure 11. Through a Wi-Fi connection established between our computer and the recording device, we obtained the real-time video stream with the matching overlays that created the AR experience.

#### 4.2.2. Evaluation of the Prototype

To assess the developed prototype, we designed and performed three different tests. In the first one, the objective was to verify how the marker detection technology behaves with different distances, perspectives, and camera specifications. The second one was intended to understand how fast a user could react to the representation of a virtual element, from the moment the solution detects a marker. The third evaluates, with users, the improvements that AR visual hints can bring to UAV training circuits relative to the perception of the trajectory.

**Test 1—Detection of the marker according to different distances, perspectives, and cameras**

First, to evaluate the suitability of the technology used in the implementation, we defined three test scenarios based on a marker, measuring 18.5 cm by 18.5 cm, printed on an A4 page. In the first scenario, the marker was fixed to a wall, perpendicular to the ground, in an outdoor scenario with cloudy weather. The second one was similar, but the marker was fixed onto the wall of an indoor garage with standard incandescent lights, during the night. In the third one, the marker that was fixed on the ground was overflown by a drone with its camera pointing down. These tests were used to assess the efficiency of the detection with different distances and inclinations.

**Figure 12:**
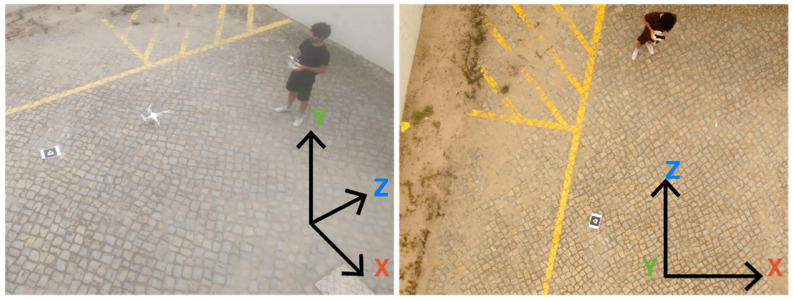
Test scenario of the pictures captured with a DJI Phantom 4 and an example of one of the pictures captured with a 7.5 m altitude.

This experiment had four sets of seven iterations with three different devices: a low-mid range smartphone Xiaomi MI A2, an out-of-the-box DJI Phantom 4 drone (Figure 12), and a system to simulate a cost-effective modularly built UAV. The cost-effective equipment used to take the pictures was a Raspberry Pi 3 and a Pi NoIR Camera V2.1 with a still resolution of 8 MP. The pictures were captured in the default size of each device and saved in the JPG format.

The smartphone and the drone captured pictures with 9 MP. Every iteration consisted of taking 10 pictures from different angles and positions, with the defined distances of 1 m, 2.5 m, 5 m, 7.5 m, 10 m, 12.5 m, and 15 m. In the case of the aerial drone footage, the distance was measured with the DJI GCS app, DJI GO 4. So, the first scenario resulted in 140 pictures, 70 captured with the Raspberry Pi and 70 with the smartphone. The second scenario resulted in 70 pictures, captured with the Raspberry Pi, and the third scenario in 70 pictures, captured with the DJI drone. These pictures were loaded to the Web AR application to validate the detection and the results are represented in Table 4.

**Figure 13:**
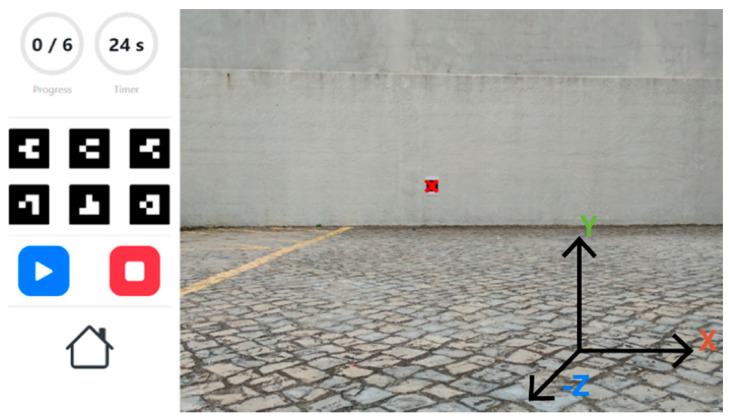
Marker found in the picture captured with the smartphone 10 m apart.

These results show the percentage of successes for each iteration case. In the case of 1 m apart, every iteration was successful, except for a picture captured by the Raspberry Pi in the garage, which presented a shadow crossing the marker. In the case of 2.5 m apart, every iteration was successful. In the case of 5 m apart, only the pictures captured by the smartphone were fully successful. In the case of the pictures captured by the Raspberry Pi outside and in the garage, the system did not detect the marker in 1 of them, both presenting a success rate of 90%. The drone presented the worst results, with only 2 detections out of 10. In the case of 7.5 m apart, both the Raspberry Pi outside and the smartphone presented a 50% success rate, whilst the Raspberry Pi in the garage shows better results, with 7 detections out of 10. The drone did not provide any detection with an altitude of 7.5 m. In the case of 10 m apart, the detection only happened in 2 pictures of the smartphone. In the cases of 12.5 m and 15 m, there was no detection. The smartphone samples were the only ones that could detect AR markers up to 10 m, whilst the samples from the DJI Phantom drone could only detect the AR markers up to 5 m. Additionally, the smartphone presented the best results for detection with the longest distance (Figure 13), with 2 true positives (TPs) in 10 samples, whilst the DJI Phantom presented the worst results, with a total of only 22 detections against 37 detections with the smartphone. The Raspberry Pi presented good results both outdoors and indoors with low luminosity.

**Figure 14:**
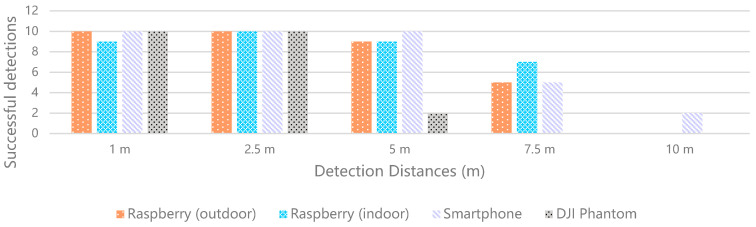
Number of detections (true positives) for distances up to 10 m in each test scenario of Test 1.

In this context and according to the performance of the detection system, the results are interpreted as follows. The true positives (TPs) are the samples with detected AR markers; the true negatives (TNs) are the samples without AR markers and no detection; the false positives (FPs) are the samples without AR markers and detection; and the false negatives (FNs) are the samples with undetected AR markers. Among the iterations, there are no samples without AR markers, which means that there are no TNs or FPs. These circumstances influence the precision and recall. The precision value is 1 for every iteration because there are no false positive samples, since it considers the TP samples relative to all the positive ones (i.e., TPs and FPs). On the other hand, the recall corresponds to the normalized success percentage that describes the results of the 10 samples for each distance, so the higher the recall, the better the performance of the system. This value considers the TP values relative to the total of relevant samples (i.e., TPs and FNs). These values are represented in Table 5, which excludes the samples of the distances of 12.5 m and 15 m, since the system did not work for those distances. The TP samples are presented in Figure 14.

Table 6 presents the average success rates for each distance, independently of the test scenario, whilst Table 7 presents the average success rates for each test scenario, independent of the distance. The analysis of these values shows that the distance with the best results was 2.5 m, whilst the test scenario with better performance was the one where the samples were captured with the smartphone. Regarding the performance of the proposed system, considering the tested characteristics, we can expect reliable results with distances of up to 5 m.

By analyzing the pictures in which the marker was not detected, we realize that shadows that partially change the color of the marker can negatively affect its segmentation and the subsequent detection. The solution is intended to work according to the frames obtained from a video feed, preferably of 30 frames per second. This means that in a second, the algorithm can receive 30 images to process, and some can be blurred or present light artifacts, but there should also be good frames with a clean capture of the marker.

The solution works from different angles and distances, which can go up to 10 m with the smartphone. This supports the development of a cost-effective AR experience for UAVs, which can be used for pilot training or the assessment of related solutions in a dynamic virtual environment, and that saves the user from spending money on physical obstacles for flying courses. Additionally, the success of the solution will depend on the quality of the camera and size of the marker. In this case, for cost-effective equipment, the identification of the marker is achievable from 7.5 m, which suggests that with a better camera and bigger marker, the solution would work from a longer distance.

**Test 2—Measurement of the user reaction time after the detection of a marker**

Next, to evaluate if the use of the solution was plausible in real time, we defined a test scenario consisting of a simple AR web application, a laptop, and a printed marker. This test was designed to measure how long a user would take to react after the system identified a marker and displayed the respective virtual element.

In the web application, we included the basic AR functionalities required in the development of the solution, which are the detection of a marker and the representation of a 3D model. In this application, we added a button to register timing in the event of a click. The time interval (∆t) considered for the measurements starts with the event triggered by the AR.js library when a marker is found and ends with the click of the register button, as in the following Equation (1). Each timing includes the delay from the mouse input.

(1) $$\Delta t\;=\;\left(t_{button\;click}-t_{marker\;detection}\right)\;+\;t_{input\;delays}$$

The web application was deployed locally, since the implementation of the system followed the pure front-end approach, which means that the AR part of the solution always runs on the client device. So, for these measurements, it is not relevant if the solution is deployed locally or whether it is cloud hosted. The experiment was performed in daylight conditions, using a laptop, its webcam, and a barcode marker. With the application running, we passed the marker in front of the webcam and clicked on the register button when the 3D model was presented. The samples were collected during the daytime, on a sunny day, in a room filled with natural sunlight. From the 108 registered samples, we discarded eight outliers that resulted from input errors, resulting in 100 samples, presented in Table 8, sorted from highest to lowest, divided into 5 groups of 20 samples, and rounded to 3 decimal places.

**Figure 15:**
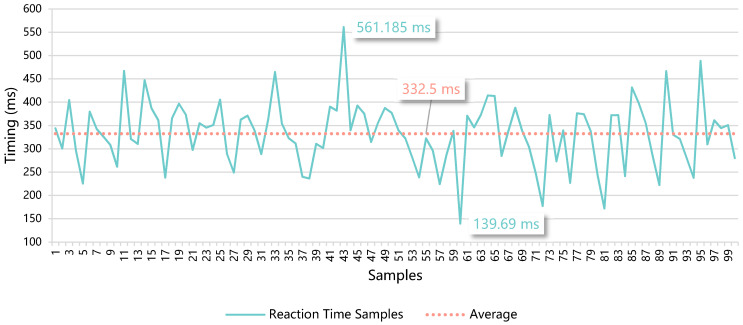
Results from Test 2, performed to measure the timings corresponding to how long a user takes to react after the detection of a marker and representation of the respective 3D model.

The average reaction time for the 100 samples was 332.5 ms, as represented in Figure 15. The median was 339.4 ms with a standard deviation of 69.2 ms. These data show an equilibrium of the timings represented by the samples relative to the average, since the median value is close. As the standard deviation shows, there is not a significant difference between the sample values and the average. This means that, on average, the event corresponding to the detection of a marker by the system until the reaction of the user based on the representation of the respective virtual element would take approximately a third of a second. This is a small interval that does not jeopardize the AR experience. The users can undergo a fluid interactive training experience in real time, with a real drone and in a real environment, which is enhanced by virtual elements presented through Web AR technologies.

**Test 3—User testing to validate AR hints as an improvement in a simulated circuit**

**Figure 16:**
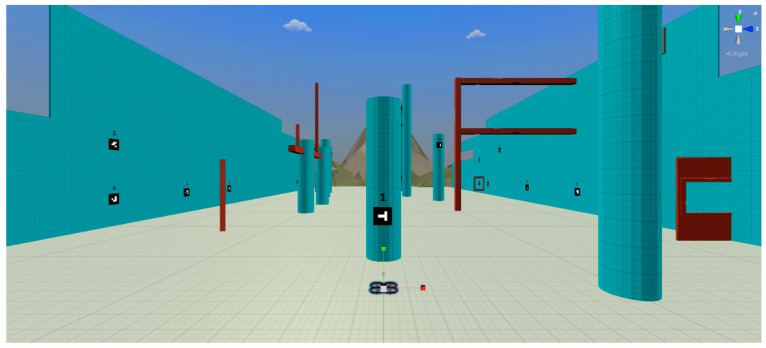
Virtual environment simulated in Unity for Test 3, with the trajectory configured for the second test scenario (i.e., level 2).

To validate the proposed training solution with users, we defined two test scenarios based on the same environment, presented in the virtual simulation created in Unity, where a quadcopter can be fully piloted through a keyboard or a gamepad, which is similar to the real UAV controllers (Figure 16). The simulator is available at the link presented in the Supplementary Materials section.

This assessment intends to validate if the addition of dynamic AR visual hints with the POIs of an unknown trajectory improves to the flight times. With that purpose, this test considers the times registered for the first time the participants complete the two configured circuits, that correspond to the two test scenarios, represented by levels 1 and 2 in the simulation. The first level corresponds to a circuit made of 26 POIs, which create a trajectory of 443.56 m. The second level corresponds to a circuit made of 26 POIs as well, which creates a trajectory of 432.42 m.

In both levels, the participants must detect the markers in the correct order, which is represented with text above the markers, in this case, from 1 to 26. When a participant detects a marker, the simulation checks if it is part of the defined trajectory, as the next POI to visit, or not. These two situations trigger an audio feedback that represents success and failure. Additionally, when the participants detect the first marker, the stopwatch available in the simulation starts counting. When the participants arrive at the 26th marker, the recorded time is presented on the screen in the format “00:00:00:000”, showing hours, minutes, seconds, and milliseconds. The data were converted to minutes and rounded to three decimal places, to facilitate their presentation and interpretation.

**Figure 17:**
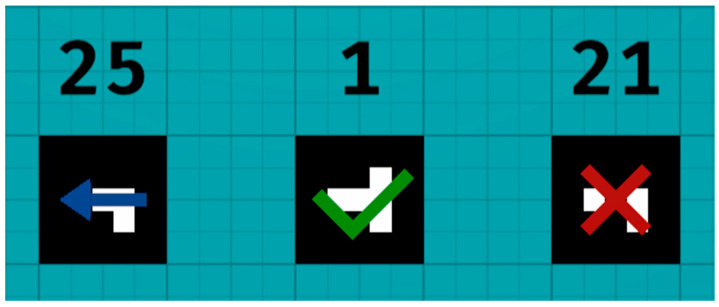
AR visual hints presented in the training simulation with an orthographic rendering.

The difference between the two levels is the absence or presence of the visual hints. Level one only presents the markers with a numeral indicator on top, representing the order of the trajectory. As the solution proposed in this paper, level 2 simulates the addition of AR visual hints when the drone gets close to the markers, as presented in Figure 17. The visual hints are arrows pointing at the next marker, a check sign if the marker was already validated, and a cross if the detection does not follow the defined trajectory. To prevent the results of the second level from being influenced by an improvement in the control skill, we provided a training level with the same environment, so that users can get used to the control before starting the experience.

As for the simulated quadcopter, it flies with a maximum speed of 6 m/s, either when moving along its horizontal plane or increasing and decreasing its altitude, and it rotates 90 degrees per second along its vertical axis, to perform turns leftwards and rightwards.

**Figure 18:**
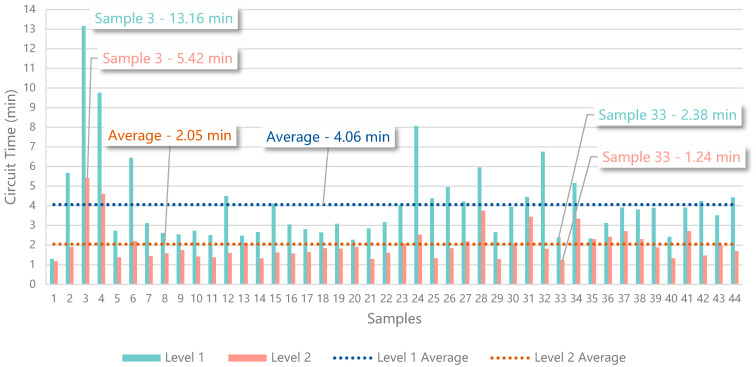
Results from Test 3, performed to measure the influence of AR visual hints in decreasing the circuit time for users that are controlling a UAV for the first time in an unknown trajectory.

This evaluation was performed by 44 participants, 31 men and 13 women, with ages from 18 to 58. After the experience, each participant answered a Google Forms survey to upload the results, which are presented in Table 9 and Figure 18, where the first sample corresponds to the control values obtained by the authors that built the trajectory, for comparison purposes (i.e., 1.298 min in level 1 and 1.172 min in level 2).

The third sample had the highest values (i.e., 13.162 min in level 1 and 5.416 min in level 2), whilst the sample with the lowest values, besides the control sample, was sample 33 (i.e., 2.368 min in level 1 and 1.233 min in level 2). There are 5 samples that can be identified as outliers, which correspond to users with less dexterity and less experience in control interfaces (i.e., samples 3, 4, 6, 24, 32). Samples 13 (i.e., 2.480 min in level 1 and 2.085 min in level 2) and 35 (i.e., 2.339 min in level 1 and 2.303 min in level 2) describe the results from two participants that gave up from level 1, due to not being able to find the marker identified by the number 4, then finished level 2 and returned to finish level 1, failing to comply with the test rules. The average from level 1 is 4.061 min (i.e., 4 min, 36 s, and 600 ms), whilst the average from level 2 is 2.052 min (i.e., 2 min, 3 s, and 120 ms). The comparison of the average values shows that the participants were 1.979 times faster traveling the trajectory of level 2, which includes the simulation of the AR visual hints. From the 44 participants, 5 reported that having to search for the trajectory in level 1 was annoying, but then enjoyed flying in level 2, following the AR visual hints. Additionally, 8 participants complained about the controls not being intuitive, which is a common feeling among users that are not used to the piloting of UAVs.

The results greatly support the use of AR visual hints to improve the performance of the participants learning to fly along a trajectory. The addition of the visual hints (i.e., arrow, check, and cross) in level 2 substantially decreased the time required to finish a similar trajectory, within the same environment configuration.

## 5. Conclusions and Future Work

This study resulted in the proposal of a solution that enables a novel AR application for UAV pilot training and assessment, considering the remote control of the vehicle from a non immersive exocentric point of view. With this solution, the user can decide how to control a UAV based on the images captured by its camera, augmented with virtual content, and displayed on a screen. Using Web AR technologies to develop the prototype, it was possible to create a cloud-hosted AR experience theoretically compatible with all devices supporting a camera and a web browser. With this solution, it is possible to create several digital scenarios in the same real-world environment, each one with challenging paths, new symbology, and tasks. All of this can be used for learning and to improve UAV piloting skills, as well as to help developers and researchers create the required test environments. This study can help mitigate some of the problems found in HDI resulting from the technophobia related to crashes and the difficult and dangerous process of creating test scenarios with obstacles placed in high places. In the end, the proposal helps pilots and researchers and provides an inclusive solution to improve the engagement of any type of user, from neophytes to enthusiasts, in UAV-related activities. This proposal is particularly useful for researchers because it greatly improves the approach of creating countless trajectories for different test scenarios after a single placement of AR markers in a real environment, which can be configured with a new meaning.

This proposal, focusing on UAV control, can also be applied in other areas, such as navigation for outdoor digital guided tours. This novel approach presents advantages over the traditional methods of AR and UAV training, which is usually performed in entirely virtual simulators. One of them is to pilot real UAVs with real controllers, which delivers an authentic experience instead of a simulation. This solution, based on trendy and emergent technologies, is easily available thanks to its multiplatform approach. It is cost-effective and establishes an easy-to-use solution to potentiate user training through an AR representation of dynamic courses made of visual markers. The use of virtual obstacles reduces the fear of crashing the aircraft, which can improve the engagement of novice users. These characteristics intend to potentiate the engagement of the users and, therefore, help in the mitigation of the exclusion that still exists in trendy technologies, related to technophobia issues or approaches limited to expensive equipment that is usually not available for the common user.

The solution was implemented and the resulting prototype underwent three different tests. The first test allowed us to validate the marker detection technology in different environments, with different light conditions and with different cameras. The results confirmed the capacity to detect a marker from a distance of 10 m, though shorter distances had greater success, even from different perspectives. As the second test illustrated, the solution allows users to take a third of a second on average to react to a stimulus of a virtual element represented over a marker after its detection. These results validate the use of this solution in real time without jeopardizing the experience of training with a real drone in a real environment that is enhanced with virtual elements through Web AR technologies. This enhancement is verified with the results of the third test, based on a virtual simulation experienced by 44 participants, that showed a significant decrease in the time required to fly along a circuit when using AR visual hints.

As part of future work, the prototype should be improved and integrated into out-of-the-box drones, such as the DJI Phantom and other DIY vehicles included in this study. Another future task should be to perform more user-experience studies in real scenarios and real UAVs to understand the impact of the proposed solution with realistic characteristics, to validate the results obtained in Test 3 through the simulation. A study should be conducted with a group of subjects with different levels of UAV piloting skills to determine their individual improvement rate, based on how long they take to finish several AR trajectories, which can be measured through the already developed marker detection system. Additionally, a questionnaire should be completed by the participants of the test experience to evaluate the quality and usability of the solution through the Likert scale.

## Figures and Tables

**Table 1:** Limitations found in the presented Web Augmented Reality development approaches.

Approach	Limitation
Pure front-end	JavaScript performance
Browser kernel-based extension	Dedicated cross-platform development
Browser + cloud	Increased latency

**Table 2:** Differences between the strategies depicted and included in the proposed solution, which are the treasure hunt and the obstacle course modes.

Modes	Treasure Hunt	Obstacle Course
Markers	Arrows pointing the way	Different obstacles, e.g., cones, poles, hoops, gates
Tasks	Follow the trajectory	Dodge the hazards
Goals	Learn the basic flight mechanics, improve maneuverability and capacity to follow well-defined paths as fast as possible	Improve reaction time and decision capacities for trajectory planning

**Table 3:** Meanings of the four statuses that define the markers during the AR experience.

States of the Markers	Meaning
Next	Next marker in the sequence that must be detected to successfully continue the trajectory (i.e., activate the next point of interest (POI) to detect) until reaching the last one
Current	First or last marker to be detected successfully (i.e., complying with the sequence)
Validated	All the markers that were already detected successfully and are not the current one
Invalid	All the markers that are yet to be detected and are not the next one

**Table 4:** Success percentage for each test scenario and iteration of 10 pictures per distance.

Resolution	8 MP	8 MP	9 MP	9 MP
Device	Raspberry Pi (Outdoor)	Raspberry Pi (Indoor)	Smartphone	DJI Phantom
Distance (m)	Success Percentage (%)			
**1**	100	90	100	100
**2.5**	100	100	100	100
**5**	90	90	100	20
**7.5**	50	70	50	0
**10**	0	0	20	0
**12.5**	0	0	0	0
**15**	0	0	0	0

**Table 5:** Compilation of the results from Test 1, categorized by test scenario and distance.

Iteration	Distances	Samples	TP	FN	Precision	Recall
**Raspberry Pi (outdoor)**	1 m	10	10	0	1	1
	2.5 m	10	10	0	1	1
	5 m	10	9	1	1	0.9
	7.5 m	10	5	5	1	0.5
	10 m	10	0	10	1	0
**Raspberry Pi (indoor)**	1 m	10	9	1	1	0.9
	2.5 m	10	10	0	1	1
	5 m	10	9	1	1	0.9
	7.5 m	10	7	3	1	0.7
	10 m	10	0	10	1	0
**Smartphone**	1 m	10	10	0	1	1
	2.5 m	10	10	0	1	1
	5 m	10	10	0	1	1
	7.5 m	10	5	5	1	0.5
	10 m	10	2	8	1	0,2
**DJI Phantom**	1 m	10	10	0	1	1
	2.5 m	10	10	0	1	1
	5 m	10	2	8	1	0.2
	7.5 m	10	0	10	1	0
	10 m	10	0	10	1	0

**Table 6:** Average success rate per distance, considering every test scenario up to 10 m.

Distance	1 m	2.5 m	5 m	7.5 m	10 m
**Average Detection Rate (%)**	97.5	100	75	42.5	5

**Table 7:** Average success rate per test scenario, considering distances up to 10 m.

Iteration	Raspberry Pi (outdoor)	Raspberry Pi (indoor)	Smartphone	DJI Phantom
**Average Detection Rate (%)**	68	70	74	44

**Table 8:** Samples collected for Test 2, which represent the user reaction timing after the detection of a marker, sorted from highest to lowest, divided into 5 groups of 20 samples.

Iterations	1	2	3	4	5
**Samples (ms)**	561.185	377.315	351.030	321.235	280.190
	488.670	376.290	350.870	321.085	279.360
	467.065	375.480	345.990	314.610	273.150
	466.720	374.230	345.475	311.430	261.650
	464.870	373.220	344.490	310.755	249.020
	447.255	373.210	344.130	310.560	247.145
	431.710	372.585	342.855	309.635	243.690
	414.550	372.370	340.035	308.445	241.420
	413.250	372.210	339.765	304.205	240.200
	405.470	371.215	339.545	301.465	238.835
	404.725	371.055	339.290	300.610	238.175
	397.930	365.675	339.250	297.475	237.760
	396.760	362.910	338.350	296.170	236.390
	392.840	362.665	338.290	295.955	226.865
	390.475	361.235	337.970	288.980	225.590
	388.125	361.175	330.140	288.705	224.295
	387.650	355.680	326.335	287.695	222.205
	386.955	355.575	322.805	286.625	177.270
	381.910	355.010	322.345	284.770	171.795
	379.745	354.070	322.280	281.035	139.690

**Table 9:** Samples collected for Test 3, performed to measure the influence of AR visual hints in decreasing the circuit time for users that are controlling a UAV for the first time in an unknown trajectory.

Samples	Level 1	Level 1 (min)	Level 2	Level 2 (min)
1	00:01:17:899	1.298	00:01:10:299	1.172
2	00:05:40:381	5.673	00:01:54:356	1.906
3	00:13:09:734	13.162	00:05:24:971	5.416
4	00:09:45:697	9.762	00:04:36:304	4.605
5	00:02:43:412	2.724	00:01:22:560	1.376
6	00:06:26:640	6.444	00:02:12:066	2.211
7	00:03:06:613	3.110	00:01:26:213	1.437
8	00:02:37:000	2.617	00:01:35:000	1.583
9	00:02:32:150	2.536	00:01:45:100	1.752
10	00:02:43:868	2.731	00:01:24:806	1.413
11	00:02:30:241	2.504	00:01:22:718	1.379
12	00:04:30:188	4.503	00:01:35:962	1.599
13	00:02:28:771	2.480	00:02:05:105	2.085
14	00:02:39:340	2.656	00:01:19:218	1.320
15	00:04:06:262	4.104	00:01:37:698	1.628
16	00:03:03:140	3.052	00:01:34:315	1.572
17	00:02:48:055	2.809	00:01:37:989	1.633
18	00:02:38:331	2.639	00:01:50:664	1.844
19	00:03:05:145	3.086	00:01:49:525	1.825
20	00:02:16:183	2.270	00:01:54:621	1.910
21	00:02:50:630	2.844	00:01:17:510	1.292
22	00:03:10:342	3.172	00:01:36:537	1.609
23	00:04:01:922	4.032	00:02:03:840	2.064
24	00:08:04:188	8.070	00:02:31:843	2.531
25	00:04:22:139	4.369	00:01:19:899	1.332
26	00:04:56:063	4.944	00:01:50:217	1.837
27	00:04:13:000	4.217	00:02:10:962	2.183
28	00:05:57:604	5.960	00:03:45:269	3.754
29	00:02:39:326	2.655	00:01:16:916	1.282
30	00:03:56:111	3.935	00:02:03:832	2.064
31	00:04:27:000	4.450	00:03:27:000	3.450
32	00:06:45:412	6.757	00:01:48:865	1.814
33	00:02:22:089	2.382	00:01:14:002	1.237
34	00:05:09:602	5.160	00:03:20:769	3.346
35	00:02:20:315	2.339	00:02:18:199	2.303
36	00:03:07:470	3.125	00:02:25:320	2.422
37	00:03:54:436	3.907	00:02:42:334	2.706
38	00:03:48.573	3.810	00:02:18.002	2.303
39	00:03:54:000	3.900	00:01:53:000	1.883
40	00:02:24:951	2.416	00:01:19:944	1.332
41	00:03:54:436	3.907	00:02:42:334	2.706
42	00:04:14:512	4.242	00:01:28:021	1.470
43	00:03:30:778	3.513	00:01:58:836	1.981
44	00:04:25:754	4.429	00:01:41:977	1.700

## Data Availability

Not Applicable.

## Supplementary Materials

The simulator used to perform Test 3 with users is available online at https://puzzlepack.itch.io/webar-training-simulator.
